# COVID 19: Psychological Effects and Associated Factors in Mexican Nurses

**DOI:** 10.1017/dmp.2020.495

**Published:** 2020-12-22

**Authors:** Nadia Yanet Cortés-Álvarez, César Rubén Vuelvas-Olmos

**Affiliations:** 1 Department of Nursing and Midwifery, Division of Natural and Exact Sciences, University of Guanajuato, Guanajuato, Mexico; 2 PhD Medical Sciences Program, School of Medicine, University of Colima, Colima, Mexico

**Keywords:** COVID-19, nurses, traumatic distress, psychological distress, emotional exhaustion

## Abstract

**Objectives:**

The present study examined the psychological effects and identify factors associated with worse outcomes, during the coronavirus disease 2019 (COVID-19) outbreak in Mexican nurses involved in fighting against COVID-19.

**Methods:**

An anonymous online questionnaire was applied through an online survey, which collected information regarding basic information, traumatic distress response (IES-R scale), emotional exhaustion (MBI-EE), and psychological distress (K10 scale).

**Results:**

Results showed that 46.72% of nurses reported moderate-severe traumatic distress response, 42.40% of nurses evidenced a high level of emotional exhaustion, and 41.78% showed moderate-severe psychological distress. Nurses who have >2 children, an increase in working hours due to COVID-19, increase in tobacco and alcohol consumption, and presence of a confirmed and suspected case of COVID-19 in their workplace showed worse outcomes.

**Conclusions:**

These findings demonstrate that a large portion of nurses in Mexico is suffering from psychological disturbances due to the COVID-19 outbreak. In the face of a health crisis, not seen in several years in Mexico, the proper psychological well-being of the nursing staff at this vulnerable time is essential.

Coronavirus disease 2019 (COVID-19), an infectious disease caused by the severe acute respiratory syndrome coronavirus 2 (SARS-CoV-2) virus, originated in the city of Wuhan, China, on December, 2019.^1^ Since then, rapid community, regional, and international spread has occurred, with an exponential growth in cases and deaths, becoming a Public Health Emergency of International Concern.^2^ The COVID-19 outbreak was confirmed to have reached Mexico on February 28, and on March 30, the Mexican government declared a national health emergency.^3^ To date (September 10, 2020), 681,321 COVID-19 cases and 70,818 COVID-19-related deaths have occurred^4^; with these data, Mexico is fourth in deaths from COVID-19 in the world.^5^ The vast majority of documented deaths have occurred within hospitals, leading to the psychological impacts on health-care workers.^6^

In the face of a health crisis, not seen in several years in Mexico, COVID-19 is putting health workers, including nursing staff under intense pressure. Historically, nurses have always played an important role in infection prevention, infection control, isolation, containment, and public health, as initially advocated by Florence Nightingale.^7^ Nurses on the front line in this event are working under enormous pressure to battle this life-threatening viral infection, showing the commitment and compassion that nurses do everywhere.^8^

Worryingly, many of these nurses and health-care professionals are not only fighting the virus, they are also fighting the humanitarian crisis with limited protective supplies.^7,9^ Lack of protective supplies hinders adherence to guidelines for the protection of frontline health-care workers, provided by the World Health Organization (WHO), putting their own lives on the line. In addition, personal protective equipment, such as masks, provides protection of mental health in some countries.^10-12^ At the time of writing this article, COVID-19 has already claimed the lives of 1320 health-care professionals,^13^ and 46,000 have been infected.^14^ In fact, actually Mexico is the country that registers the most deaths of health workers from COVID-19.^15^

It has been identified that, when nurses are exposed to working environments with high job demands and low resources, higher job stress and greater physical and psychological stress symptoms may adversely affect health and well-being.^16,17^ Sadly, despite the efforts of the nursing staff to safeguard the life of the population, nurses have been stigmatized as vectors of contagion and have been assaulted, abused, and marginalized. They have even been prevented from using public transport due to their occupation. Many nurses no longer wear their uniforms while traveling to or from work for fear of being hurt.^18,19^ Maintaining the mental health of nursing staff is essential to control infectious diseases.^20^

While the scientific community and the WHO are still working on many unanswered aspects of this outbreak, nurses are responding to this uncertain situation based on the limited confirmed information. At present, studies on the epidemic situation of COVID-19 mostly focused on epidemiological investigation, prevention and control, diagnosis, and treatment. Fewer studies have investigated the mental health problems of clinical medical workers during the epidemic of COVID-19.^21^ Based on our understanding, there is no information about implication on the mental health problems of Mexican nurses during the pandemic. This is especially pertinent with the uncertainty surrounding an outbreak of such unparalleled magnitude in our country. Therefore, this study aims to examine the emotional effects (emotional responses, traumatic distress response, psychological distress, and emotional exhaustion) and identify factors associated with worse outcomes, during the COVID-19 outbreak in Mexican nurses.

## Methods

### Study Design

This study used a cross-sectional design, from May 25 to June 5, 2020, in Mexico.

### Research Population and Inclusion Criteria

Participants of this study were Mexican nurses, chosen based on the following eligibility criteria: agreeing to participate in the study by approving the online informed consent form, and Mexican nurses of both genders who are involved in the front line of COVID response in Mexico.

### Sample Size and Sampling Method

An anonymous online questionnaire in Spanish was applied through an online survey platform (Google Forms, Google Inc., California USA). A snowball sampling technique was carried out to recruit participants. First, a virtual meeting was held with the head nurse of state of Colima, where the aim of the research was presented and her support was requested. The state head nurse of Colima was asked to generate a list of nurses who met the inclusion criteria; as a result, a total 35 hospital head nurses and 20 clinical nurses were obtained from the states of Colima and Jalisco. The link to the survey was sent to these nurses by means of social networks and emails. They accessed the link by means of their laptops/tablets/smartphones and sent the research invitation to different clinical nurses by social networks and emails after finishing the survey. Participants were encouraged to roll out the study to as many as possible. This study was conducted in compliance with the Norma Oficial Mexicana-012-SSA3-2012, Declaration of Helsinki and the Ethics Committee of Universidad José Martí (Approval number 2020-3).

### Measure and Instruments

At the beginning of the survey, an introduction to the research was presented and informed consent was obtained. After agreeing to be involved in the study, participants answered a range of questions, including the following:

#### Basic Information Section

Initially, participants provided their professional license number and hospital workplace, to make sure they met the inclusion criteria and avoid duplication of respondents. The data collected were about age, gender, marital status, parental status, and number of children. In addition, the survey included questions related to tobacco and alcohol consumption (before and during the COVID-19 outbreak), increase in working hours per week due to COVID-19, and the presence of a confirmed and/or suspected of COVID-19 case in their workplace.

#### Traumatic Distress Response

The Impact of Event Scale-Revised (IES-R) was used. The IES-R is a self-administered questionnaire that has been applied to identify the traumatic distress response of COVID-19 among the Mexican population,^22^ and Chinese,^23^ Filipino,^24^ and Vietnamese people.^25^ The questionnaire is composed of 22 multiple choice items. The responses are rated on a 5-point scale ranging from 0 (“not at all”) to 4 (“completely agree”). The total IES-R score was used to perform the inferential statistics, whereas for descriptive statistics the total IES-R score was divided into normal (0-23), mild (24-32), moderate (33-36), and severe psychological distress (>37).^22^

#### Emotional Exhaustion

The Maslach Burnout Inventory (MBI) is a survey that covers 3 areas: Emotional Exhaustion (EE), Depersonalization (DP), and low sense of Personal Accomplishment (PA).^26^ Because the current study sought to measure feelings of being emotionally overextended and exhausted at one’s work, this study used the 9-item Emotional Exhaustion subscale only (MBI-EE). Responses were scored on a 6-point Likert scale (ranging from 0 = never to 6 = everyday). The maximum score is 54. The total EE subscale score was tabulated into 3 tiers: high (27-54), medium (19-26), and low (0-18).^27^

#### Psychological Distress

The Kessler Psychological Distress Scale (K10) was used to assess the psychological distress of the COVID-19 outbreak. The K10 is a 10-item questionnaire that measures distress levels, using questions on anxiety and depressive symptoms that a person has experienced in the most recent 4-wk period. Response choices are based on a 5-point Likert-type scale ranging from 1 (none of the time) to 5 (all the time). Responses are summed to create a total score (range = 10-50). Based on these scores, subjects were classified as having no distress (<20), mild distress (20-24), moderate distress (25-29), and severe distress (≥30).^28^ This scale has been used previously in research related to COVID-19^29,30^ and also has been validated for use in Spanish.^31^

### Statistical Analysis

Counting data were expressed by frequency and percentage, and variable continuous were expressed as mean and standard deviation. Different simple linear regression models were used to analyze the associations of variables on traumatic distress response (IES-R), emotional exhaustion (MBI-EE), and psychological distress (K10). All statistical analyses were performed with SPSS for Windows 25.0, with 2-tailed *P* < 0.05 to be considered statistically significant.

## Results

### Basic Information

A total of 500 Mexican nurses were invited to participate in this study, finally 462 nurses accepted to participate in the study (including head nurses and clinical nurses, response rate: 92.4%). Sociodemographic characteristics of the final sample of respondents are presented in Table 1. The majority of nurses were female (77.05%), aged 18 to 29 y (44.30%), single (44.30%), with children (59.74%); of these nurses with children, most reported having only 1 child (28.57%). Regarding tobacco consumption, 22.94% nurses reported smoking, and 16.88% considered that their consumption had increased during COVID-19. Regarding alcohol consumption, 41.12% of nurses reported consuming alcohol, and of these, 14.72% considered that their consumption had increased during COVID-19. Finally, 47.18% reported an increase in their working hours per week due to COVID-19, 88.31% had been in contact with a suspected case, and 77.32% with a confirmed case of COVID-19.

**Table 1.** Sample characteristics

	*N*	Percent
Gender		
Male	106	22.95
Female	356	77.05
Age (years)		
18 to 29	180	38.96
30 to 39	186	40.25
40 to 49	84	18.18
50 or more	14	3.03
Marital status		
Single	105	44.30
Married common-law	41	17.30
Married	74	31.22
Divorced	15	6.33
Widow	2	0.84
Parental status		
With children	276	59.74
No children	186	40.26
Number of children		
0	186	40.26
1	94	20.34
2	132	28.57
3	44	9.52
>3	6	1.29
Smoke		
Yes	106	22.94
No	356	77.06
Increase smoking during COVID-19		
Yes	78	16.88
No	28	6.06
N/A	356	77.06
Consumption alcohol		
Yes	190	41.12
No	272	58.88
Increase working hours per week		
Yes	218	47.18
No	244	52.82
Increase consumption alcohol during COVID -19		
Yes	68	14.72
No	122	26.40
N/A	272	58.88
Contact in work with suspected case COVID-19		
Yes	408	88.31
No	54	11.69
Contact in work with confirmed case COVID-19		
Yes	348	75.32
No	114	24.68

*N* = Sample size.

### Traumatic Distress Response, Emotional Exhaustion, and Psychological Distress

The traumatic distress response from the COVID-19 outbreak was measured using the IES-R scale. Of the total respondents, 46.72% reported moderate to severe traumatic distress response, 32.29% rated mild, and 20.99% reported normal traumatic distress response. Regarding the MBI-EE subscale, used to measure emotional exhaustion, 42.40% of nurses had a high level, 31.88% showed a medium level, and 25.72% reported a low level of emotional exhaustion. Finally, according to the K10 scale, which assessed the psychological distress of the COVID-19 outbreak, 41.78% of nurses showed moderate to severe psychological distress, 26.39% reported mild level, and 31.84% showed normal psychological distress.

### Association Between Basic Information and Traumatic Distress Response, Emotional Exhaustion, and Psychological Distress

Results are shown in Table 2. Data on gender, age, marital status, parental status, tobacco and alcohol consumption were not associated with any scale used in the study. Findings indicated that nurses who have >2 children were significantly associated with higher scores on psychological distress (K10 scale; *P* = 0.041). The increase in working hours per week due to COVID-19 was significantly associated with higher scores on traumatic distress response (IES-R scale, *P* = 0.002), psychological distress (K10 scale, *P* = 0.002), and emotional exhaustion (MBI-EE scale, *P* = 0.001). Finally, the statistical analysis showed a significant association between the presence of a confirmed case of COVID-19 in their workplace with higher scores on traumatic distress response (IES-R scale; *P* = 0.010) and psychological distress (K10 scale; *P* = 0.002). The presence of a suspected case of COVID-19 in their workplace showed a significant association with higher scores on traumatic distress response (IES-R scale; *P* = 0.014) and psychological distress (K10 scale: *P* = 0.001).

**Table 2.** Association between basic information and traumatic distress response, psychological distress and emotional exhaustion among Mexican nurses during COVID-19 pandemic

	IES-R Traumatic Distress response		K-10 Psychological Distress		MBI-EE Emotional Exhaustion	
Variable	R^2^	β (95% CI)	R^2^	β (95% CI)	R^2^	β (95% CI)
Gender						
Female	.001	1.136 (−5.481 to 3.209)	.001	.065 (−3.382 to 3.511)	.012	3.347 (−.566 to 7.259)
Male		Reference		Reference		Reference
Age						
18 to 29	.021	−3.744 (−12.401 to 4.912)	.008	−.189 (−7.116 to 6.738)	.006	.544 (−7.293 to 8.382)
30 to 39		1.640 (−7.000 to 10.280)		3.043 (−3.870 to 9.957)		6.495 (−1.328 to 14.317)
40 to 49		3.119 (−6.101 to 12.339)		3.738 (−3.640 to 11.116)		4.214 (−4.133 to 12.562)
50 or more		Reference		Reference		Reference
Marital status						
Single	.001	17.224 (−3.104 to 37.551)	.001	7.629 (−8.597 to 23.854)	.001	−5.714 (−24.244 to 12.815)
Married common-law		17.427 (−3.195 to 38.049)		8.000 (−8.461 to 24.461)		−4.537 (−23.334 to 14.261)
Married		18.743 (−1.664 to 39.150)		8.297 (−7.992 to 24.586)		−4.189 (−22.791 to 14.413)
Divorced		20.167 (−1.270 to 41.604)		6.800 (−10.311 to 23.911)		−8.533 (−28.074 to 11.007)
Widow		Reference		Reference		Reference
Parental status						
With children	.011	3.108 (−.653 to 6.869)	.008	−.567 (−2.433 to 3.568)	.002	−1.123 (−2.307 to 4.553)
Not children		Reference		Reference		Reference
No. of childrens						
1	.001	2.375 (−4.055 to 8.805)	.008	3.442 (−1.658 to 8.543)	.003	2.820 (−3.040 to 8.680)
2		2.345 (.458 to 14.233)		5.698* (.235 to 11.161)		5.128 (−1.149 to 11.405)
3		3.438 (−3.143 to 10.019)		4.532* (−1.253 to 6.007)		2.517 (−3.481 to 8.515)
>3		2.451 (−3.053 to 863)		6.034* (−1.872 to 10.543)		3.356 (−2.264 to 6.102)
0		Reference		Reference		Reference
Increase working hours per week						
Yes	.065	7.493* (3.883 to 11.103)	.077	6.450* (3.602 to 9.297)	.204	12.019** (8.994 to 15.044)
No		Reference		Reference		Reference
Smoke						
Yes	.057	.396 (.049 to .742)	.071	.422 (.098 to .847)	.077	.439 (.135 to .744)
No		Reference		Reference		Reference
Increase in tobacco consumption						
Yes	.060	.770 (.581 to .959)	.051	.140 (.056 to .425)	.072	.281 (.058 to .804)
No		Reference		Reference		Reference
Consumption alcohol						
Yes	.015	−.690 (−7.467 to .086)	.019	−.278 (−.469 to -.186)	.006	−.297 (−.542 to .347)
No		Reference		Reference		Reference
Increase in alcohol consumption						
Yes	.043	.465 (−.381 to .510)	.051	.296 (.141 to .752)	.052	.369 (.022 to .572)
No		Reference		Reference		Reference
Contact with suspected case COVID-19 in workplace						
Yes	.016	5.318* (−.027 to 10.662)	.004	2.109** (−2.159 to 6.376)	.078	.758 (.063 to .852)
No		Reference		Reference		Reference
Contact with confirmed case COVID-19 in workplace						
Yes	.027	5.498* (1.334 to 9.661)	.04	5.285* (2.002 to 8.567)	.125	.651 (.066 to .735)
No		Reference		Reference		Reference

β, beta coefficient; CI, confidence interval.

*P < 0.05.

**P < 0.001.

## Discussion

The nurse’s role has long been regarded as stress-filled and emotionally exhausting based upon the physical labor, human suffering, work hours, staffing, and interpersonal relationships that are central to the work nurses do. In Mexican nurses, in recent years, the prevalence of traumatic distress responses was 10% approximately,^32^ a psychological distress of 26.4%,^31^ and the high emotional exhaustion was around 18.9% to 29.6%.^33,34^ Additionally, nurses, as the largest group of health professionals, are at the frontline of health-care system responses to pandemics such as COVID-19, making them more susceptible to complex emotional reactions and psychological distress. Therefore, the present study examined the psychological effects in Mexican nurses and identified factors associated with worse outcome during the COVID-19 outbreak. In general, the results showed that a large portion of Mexican nurses is suffering moderate-severe traumatic distress as well as moderate-severe psychological distress and a high level of emotional exhaustion. The data supporting these findings are discussed below.

First, in the present study, 46.72% of nurses rated their traumatic distress response to the outbreak as moderate to severe. Our findings are consistent with previous results in Italian nurses (43-49.3%) during COVID-19^35,36^ and similar to the relatively high rates of posttraumatic stress disorder (7-53.8%) reported in the general population during the COVID-19 pandemic in China, Spain, Italy, Iran, the United States, Turkey, Nepal, and Denmark.^37^ In addition, this study found that an increase in working hours per week due to COVID-19 and contact with a suspected and confirmed case of COVID-19 in their workplace are factors associated with higher traumatic distress response to the outbreak. Other studies report similar findings. During the H1N1 pandemic, hospital workers in high-risk work environments who felt significantly more “workload,” had significant traumatic distress responses,^38^ and frontline health-care workers who engaged in direct diagnosis, treatment, and care of patients with COVID-19 in China had higher traumatic distress response.^39^ Additionally, our results may have been due to a discouraging perspective presented by Mexican health institutions^40^ in a moment when the country and the world are being besieged by the COVID-19 pandemic.

Frontline nurses are working longer hours than usual due staff shortages; therefore, they are exposed to the highest risk of infection because of their close, frequent contact with patients. And if this was not enough, nurses are working with deficient protective supplies.^7,9^ In fact, personal protective equipment provides protection of mental health.^10,11^ Of note, in Mexico, nurses have been stigmatized as vectors of contagion and have been assaulted, abused, and marginalized.^19^ This situation requires special attention because emotional breakdowns following these disgraceful episodes could trigger common psychiatric illnesses in the short- and long-terms.^41^ Accordingly, our result is not surprising, given the traumatic nature of the situations, including deficient protective supplies, long work hours, heavy work loads, exposure to the virus, contact with people infected with COVID-19, and social stigmatization, to which nurses are exposed in their daily work during this COVID-19 outbreak.

Second, our results show that 42.40% of nurses had a high level of emotional exhaustion. In the literature, it is stated that nurses and physicians are the most at-risk group among health-care workers in terms of burnout.^42^ In Mexico, the vast majority of documented deaths have occurred within hospitals, and research has shown that frontline health-care workers may experience emotional exhaustion when they communicate death notifications.^43,44^ Also, our finding corresponds to previous studies that found that the levels of emotional exhaustion appeared higher than normative values in health-care workers during the SARS^45^ and COVID-19 outbreaks in Italy.^46^ Furthermore, our results reported that an increase in working hours per week due to COVID-19 was associated with a higher emotional exhaustion score. It has been identified that, especially in health-care workers, the intense workload and the necessity of continuing work life during the pandemic increases the emotional exhaustion related to the risks taken during the care and treatment processes of COVID-19 patients,^47^ similar to our findings. This could be because demand work stressors, such as increasing workloads and greater levels of work pressure, are positively related to rates of emotional exhaustion.^48^ This result requires special attention, because it has been shown that emotional distress is frequently associated with suboptimal patient care and professional inefficiencies along with long-lasting effects on health professionals’ health status.^49^

Finally, in our study, 41.78% of nurses showed moderate to severe psychological distress, similar to that reported in nurses from Singapore (37.4%).^50^ Research has addressed that fear of an unknown can contribute to the high level of psychological distress among people during an outbreak,^51^ including health-care staff.^52^ In the present study, the psychological distress during COVID-19 in nurses who have 2 or more children, those who reported an increase in working hours per week due to COVID-19, and contact with a suspected and confirmed case of COVID-19 in their workplace, were associated with elevated psychological distress.

Similarly, it has been identified that health workers who have 2 or more children are susceptible to depression and anxiety, likely due to being placed in a considerable dilemma, that is, caring for patients and family members and needing to avoid contact with family members.^53^ Furthermore, previous studies, which were conducted when SARS emerged, revealed that the fear of contracting disease from a patient, due to direct exposure during patient care, has been associated positively with elevated psychological distress in health-care workers^54^; similar to recent findings during the COVID-19 outbreak in Israel^55^ and China.^56^ Additionally, as we know, after confirming the outbreak of COVID-19 in Mexico and during its current increase in number of cases and deaths, the nurse workforce is taking pains to struggle with the disease in the front line and protect the health of the public.^57^ These specific situations pose considerable stress on them, stress being an important risk factor for diseases.^58^ Additionally, this stress might lead to high levels of psychological distress.^23^ Of note, it has been suggested that people who report high emotional exhaustion experience and more anxiety symptoms are more likely to develop greater psychological distress.^59-61^

## Conclusions

Together, our findings demonstrate that a large portion of nurses Mexico is suffering from psychological disturbances due to the COVID-19 outbreak. In the face of a health crisis, not seen in several years in Mexico, the proper psychological well-being of the nursing staff at this vulnerable time is essential. Nurses would benefit from greater availability of personalized mental health care from psychotherapists and psychiatrists to guide positive coping styles. Therefore, it is worthwhile to contemplate the introduction of psychological interventions online or by means of telephone, such as cognitive behavioral therapy (CBT) and mindfulness-based cognitive therapy (MBCT).

In addition, as the pandemic continues, implementation of important clinical and policy strategies that include a reasonable allocation of human resources, shorter work hours, regular rest periods, and rotating shifts, as well as infant care services for nurses’ families and an adequate supply of protective materials for nursing staff who work in high-risk jobs, will facilitate nurses’ adaptation to the antiepidemic tasks.

Several limitations of the present study must be addressed. First, compared with face-to-face interviews, self-reporting online questionnaires have certain limitations related to the physical presence of the interviewer and opportunity to clarify the questions/answers. Second, the use of an online survey tool with snowball sampling produced a large data set, but it cannot be considered random or representative; therefore, results may not be generalizable. Last, the survey included no questions related to supply of face masks, and there could be a cultural difference in face mask use and effect on mental health. Notwithstanding the above limitations, this study provides invaluable information on the emotional responses in Mexican nurses during the COVID-19 outbreak, and might set the basis for future studies.
